# Prospective Evaluation of Response Outcomes of Neoadjuvant Chemotherapy in Locally Advanced Breast Cancer

**DOI:** 10.7759/cureus.21831

**Published:** 2022-02-02

**Authors:** Ashok A., Nandkishor Sopanrao Sude, Rakesh B.A., Venkata Pavan Kumar Karanam

**Affiliations:** 1 Plastic Surgery, Patna Medical College, Patna, IND; 2 General Surgery, ESIC Medical College Hospital, Hyderabad, IND; 3 General Surgery, Dr. Moopen's Wayanad Institute of Medical Sciences, Wayanad, IND

**Keywords:** margin positivity, complete clinical response, neo-adjuvant chemotherapy, breast cancer, locally advanced breast-cancer

## Abstract

Introduction

Breast cancer is a global health problem, with more than 1 million cases of breast cancer diagnosed worldwide each year, and is the most common cancer among Indian women. Locally advanced breast cancer (LABC) accounts for 10-20% in the Western world while in India it accounts for 40-50% of all cases. Locally advanced breast cancer is a very common clinical scenario especially in developing countries possibly due to various factors like lack of education and poor socioeconomic status. Women with the locally advanced disease require multimodality therapy and coordinated treatment planning. This study aimed to prospectively study the clinical profile of the LABC patients presenting to our institute and also to evaluate the role of neoadjuvant chemotherapy in downstaging the tumor.

Materials and Methods

Seventy patients diagnosed with locally advanced breast cancer were enrolled in this prospective study. After thorough preoperative workup, patients were either taken up for upfront surgery or neoadjuvant chemotherapy followed by surgery. Post chemotherapy clinical response of the tumor and postoperative histopathological evaluation of the specimen was performed.

Results

The mean age of the patients in our study was 45 years. Out of 70 patients, 18 underwent upfront surgery, and 52 received neoadjuvant chemotherapy followed by surgery. A total of 44 cases had a clinical response to chemotherapy with 9% having a complete response. The incidence of margin positivity in the postoperative specimen was significantly lower in patients who received neoadjuvant chemotherapy.

Conclusion

Locally advanced breast cancer accounted for the predominant number of breast cancer patients mostly females in their middle age. Neoadjuvant chemotherapy was effective in downstaging the tumor in the majority of cases, although complete clinical response was lower in our study. The rate of margin positivity in mastectomy specimens can also be reduced if chemotherapy is considered prior to mastectomy.

## Introduction

Changing epidemiological trends across different population-based cancer registries in India are showing increasing trends for breast cancer-related incidence and mortality, and breast carcinoma is now the most common cancer affecting women in India [1,2]. The age-adjusted incidence rate is as high as 25.8 per 100,000 women, and the mortality is 12.7 per 100,000 women among the Indian population [2]. Between 1990 and 2016, there has been a 40% increase in the incidence rate, which indicates the rising disease burden [3]. In India, the average age of the high-risk group is 43-46 years unlike in the Western world where women aged 53-57 years are more prone to breast cancer [4].

Most of the Indian patients, at their initial evaluation, present to the clinicians at the advanced stages of the disease. Locally advanced breast cancer (LABC), an advanced form of the disease, constitute 40-45% of new breast cancer cases in India [5-6]. This is in contrast to the Western population, where only 10-20% of all breast cancer patients present as LABC [7]. Locally advanced breast cancer is a very common clinical scenario especially in developing countries possibly due to various factors like lack of education, lack of awareness among the population regarding cancer, lack of community screening programs, personal and social stigma, societal taboos pertaining to cancer, and poor socio-economic status [8].

LABC includes a heterogeneous group of breast tumors with an extensive locoregional spread that may be operable or inoperable without any clinicoradiological evidence of metastasis. According to the eighth edition of the American Joint Committee on Cancer (AJCC) cancer staging manual, T3 tumors (>5 cm), with or without skin or chest wall involvement, alone or together, inflammatory breast cancers (IBCs), matted ipsilateral and/or fixed axillary lymph nodes, ipsilateral supraclavicular lymph nodes (SCLNs), and/or internal mammary lymph nodes without distant metastases are classified as LABC [9].

According to the American Cancer Society’s recent statistics, the incidence rate for local-stage breast cancer increased by 1.1% per year, in contrast to an annual decline of 0.8% for regional disease, which may reflect a shift toward an earlier stage at diagnosis [10]. The overall 5-year breast cancer survival rate was 75% for stage III compared to 98% for stage I, signifying stage-wise variation in survival. A cumulative analysis of clinical trials recently concluded that neoadjuvant chemotherapy is effective in terms of survival and distant recurrence.

Management of LABC poses a challenge to the treating physician and the management strategy of LABC has historically evolved from a single to a multimodal approach. Neoadjuvant chemotherapy is often the first-line treatment offered to patients with LABC. Many studies have shown that neoadjuvant systemic therapy aids in therapeutic decision-making by assessing the pathological tumor response, better local control of tumor, and survival [11-13]. We intended to prospectively study the clinical profile of the LABC patients presenting to our institute and also to evaluate the role of neoadjuvant chemotherapy in downstaging the tumor.

## Materials and methods

This is a prospective observational study conducted at ESI Medical College (ESIMC) and Post Graduate Institute of Medical Science and Research (PGIMSR), Bangalore, India for a two-year period starting from 2014. It was approved by ESIMC PGIMSR Institutional Ethics Committee (No. 532/L/11/12/SYNP/PG/RGUHS/12-13/ESI-PGIMSR/Est.). Patients with locally advanced breast cancer belonging to Stage IIB(T3N0), IIIA, IIIB, and IIIC were included in this study. Exclusion criteria were male breast cancer, pregnant women, all cases with distant metastasis. Seventy consecutive patients who were admitted to our department with breast cancer and satisfying inclusion and exclusion criteria were recruited in the study. Informed consent was obtained from all the patients.

Aims and objectives

The study aimed to evaluate the role of neoadjuvant chemotherapy in downstaging the tumor prior to surgical management. Another objective was to study the effect of neoadjuvant chemotherapy on the margin status of the postoperative specimen.

Procedural methodology

Patients who presented to our department and satisfying criteria were evaluated as per National Comprehensive Cancer Network (NCCN) guidelines which included: history and physical examination, diagnostic mammogram and ultrasound as necessary, trucut biopsy for diagnosis and estrogen receptor (ER) status, progesterone receptor (PR) status, and HER2/neu (human epidermal growth factor receptor 2) receptor status, genetic and fertility counseling for hereditary cancers and premenopausal women. Metastatic workup (if signs and symptoms are present) included chest and abdomen CT, bone scan, PET CT (optional). An index core biopsy was performed in all patients to record the following histopathological features of the tumor type, estrogen receptor (ER) status, progesterone receptor (PR) status, and HER2/neu receptor status. 

Out of 70 patients, 18 (stage IIB, IIIA) were considered for upfront surgery (modified radical mastectomy ) followed by chemotherapy and radiotherapy depending on pathological staging based on the operability of the disease and patients' preference after obtaining consent. Histopathology of the specimen was recorded with emphasis on margin positivity, histopathology staging, and immunohistochemistry (IHC). The remaining 52 patients received an anthracycline-based chemotherapy regimen with 5-fluorouracil, adriamycin or epirubicin, and cyclophosphamide (FAC/FEC) every 3 weeks during -6 cycles before surgery. Tumor measurements at the baseline and after the final cycle of neoadjuvant therapy were recorded by physical examination. Although CT and MRI are best in evaluating the response, due to cost considerations and patient willingness, the response was measured clinically using caliper and tape and radiologically by USG following each cycle of chemotherapy

Clinical response was assessed using RECIST criteria by measuring tumor size and node size after neoadjuvant chemotherapy. RECIST 1.16 [14] utilized the following classifications for therapeutic response: complete response (CR), primary tumor disappearance; partial response (PR), 30% or greater decrease in the longest diameter of the primary tumor; progressive disease (PD), 20% or greater increase in longest diameter of the primary tumor; stable disease (SD), tumors that did not show either sufficient shrinkage to be classified as PR or sufficient increase to be classified as PD.

Post chemotherapy, all the patients underwent modified radical mastectomy with axillary lymph node dissection (level I, II). Histopathology of the specimen was recorded with emphasis on margin positivity, histopathology staging, and IHC. We evaluated the role of neoadjuvant chemotherapy in downstaging the tumor and achieving negative margin status. Response to chemotherapy was further evaluated based on IHC, stage nodal status.

Statistical analysis

The data were entered and analyzed using IBM SPSS Statistics for Windows, version 27.0 (IBM Corp., Armonk, NY, USA). The frequencies and percentages of all variables were computed. A Chi-square (χ2) test was used to analyze the statistical association of various variables in the study. P-value less than 0.05 was considered as statistically significant for comparative analysis.

## Results

A total of 70 patients were recruited in this study. In this study, the median age of the patients was 45 years (range 25 to 68 years), the median size of the initial tumor was 6 cm (range 2 to 9 cm). Out of 70 patients, 36 (51%) were postmenopausal women. As per the tumor, node, metastasis (TNM) staging, 66% belonged to Stage IIIA and IIIB. Predominant histopathology observed was infiltrative ductal carcinoma (81%). Receptor study helped to gain an insight into the behavior of tumor with 63% of cases having PR negative status, ER and HER2/neu had almost equal distribution. Triple-negative cancers constituted 31% of cases. The distribution of various general characteristics among the study population is presented in Table 1.

**Table 1:** Distribution of various clinicopathological characteristics of study population. ER : estrogen receptor, PR : progesterone receptor, IDC : Infiltrative ductal carcinoma, ILC : Infiltrative lobular carcinoma, HER2 : human epidermal growth factor receptor 2

Characteristics			Frequency (n)	Percentage (%)
Age(yrs)	<50		49	70
	≥50		21	30
Menopausal status	Pre-menopausal		34	49
	Postmenopausal		36	51
Stage	Stage IIB		14	20
	Stage IIIA		23	33
	Stage IIIB		23	33
	Stage IIIC		10	14
Histology	IDC		57	81
	ILC		11	16
	Others(papillary, medullary)		2	3
ER	Positive		32	46
	Negative		38	54
PR	Positive		26	37
	Negative		44	63
HER2	Positive		35	50
	Negative		35	50

Of the 70 patients in our study, 18 (26%) patients underwent upfront surgery, and the remaining 52 (74%) patients received neoadjuvant chemotherapy followed by surgery. All patients received neoadjuvant chemotherapy with anthracyclines: FEC100 (doxorubicin, 5 Fluorouracil, cyclophosphamide), the median number of cycles was three (extremes 2 to 6), median delay from the last cycle of chemotherapy and surgery was 36 days. Out of 52 patients, clinical response was observed in 44 patients. Complete clinical response was observed in five (9%) patients. Eight (17%) patients were nonresponders (stable and progressive disease). The post-neoadjuvant chemotherapy (NACT) clinical response in breast tumor according to RECIST criteria is depicted in Table 2.

**Table 2:** Post-NACT clinical response rate. NACT : Neoadjuvant chemotherapy

Clinical response	Frequency	percentage
Complete response	5	9
Partial response	39	74
Stable disease	7	15
Progressive disease	1	2
Total	52	100

Clinical response rate with respect to age, stage of presentation, histopathology, and receptor status and their statistical association is depicted in Table 3. In comparison to the response rate with respect to tumor histology, invasive ductal carcinoma has a significantly better response over other types (p<0.05). No statistically significant response was observed when comparing the response rates with respect to other factors. Response with respect to receptor status was not statistically significant individually but 98% of triple-negative cancers responded well to chemotherapy.

**Table 3:** Post-NACT clinical response rate compared with various variables. ER : estrogen receptor, PR : progesterone receptor, IDC : Infiltrative ductal carcinoma, ILC : Infiltrative lobular carcinoma, HER2 : human epidermal growth factor receptor 2, NACT : neoadjuvant chemotherapy

		Complete response	Partial response	Stable disease	Progressive disease	Total
Age (P=0.87)	<50 years	3	23	4	1	31
	>50 years	2	16	3	0	21
ER status (P=0.68 )	Positive	2	18	4	1	25
	negative	3	21	3	0	27
PR status (P=0.28)	Positive	1	13	4	1	19
	negative	4	26	3	0	33
HER2/neu (P=0.29)	Positive	3	16	5	1	25
	negative	2	23	2	0	27
Histology (P<0.05)	IDC	4	32	6	0	42
	ILC	1	7	0	1	9
	Others	0	0	1	0	1
Stage (P=0.09)	IIB	2	4	1	0	7
	IIIA	3	12	0	1	16
	IIIB	0	13	5	0	18
	IIIC	0	10	1	0	11

Pre-NACT and post-NACT nodal status is depicted in Table 4. Out of 52 patients who received NACT, 20 had N0 nodal status. Out of 32 patients with the nodal disease who received chemotherapy 21 cases showed a response; CR in 53% of cases, PR in 13% of cases, and 34% cases were nonresponders. Nodal clinical response to NACT was not statistically significant.

**Table 4:** Comparison of pre-NACT vs post-NACT nodal status. NACT : Neoadjuvant chemotherapy.

		Post-NACT nodal stage				Total
		N0	N1	N2	N3	
Pre -NACT nodal stage	N0	20	0	0	0	20 (38.4%)
	N1	14	0	0	0	14 (27%)
	N2	3	3	2	0	8 (15.4%)
	N3	0	1	0	9	10 19.2%)
Total		37 ( 71.1%)	4 (7.7%)	2 (3.8%)	9 (17.4%)	52 (100%)

Out of 18 cases that underwent upfront surgery, four cases had margin positivity compared to one case in the group receiving neoadjuvant chemotherapy. A high rate of margin positivity was seen in cases undergoing upfront surgery compared to cases receiving NACT (p<0.04) (Table 5). However, in this study, no complete pathologic response in the surgical excision specimen was seen as compared to complete clinical response.

**Table 5:** Comparison of margin status between upfront surgery and NACT groups. NACT : Neoadjuvant chemotherapy.

	Margin positive	Margin negative	Total
Upfront surgery group	4	14	18
NACT group	1	51	52
P<0.04			

## Discussion

Neoadjuvant chemotherapy has the advantage of down-staging the tumor in patients with LABC. Despite LABC being the most common type of presentation among Indian women with breast cancer, there is a paucity of literature pertaining to the outcome of NACT in LABC in India. This study aimed to assess the effect of NACT in patients with LABC at ESIMC PGIMSR.

In our study, 45 years was the median age of the patient cohort. This finding is consistent with other studies like Raina et al. [15] with the reported median age of 47 years and Min et al. [16] with 49 years as the median age of presentation. Chin et al. [17] study shows a median age of 52 years in the Jamaican population. 51% of our patients were postmenopausal, which is similar to studies by Yadav et al. [18], Chen et al. [19]. In this study, predominantly cases belonged to stage IIIA, IIIB, 33% each which is comparable to other studies - stage IIB (T3 N0) was included in the study as the tumor size was more than 8 cm in the majority of the cases. Invasive ductal carcinoma was noted in 81% of the patients which is slightly lower when compared to other studies [20-22]. 11% of cases had invasive lobular carcinoma which is higher than other studies in locally advanced breast cancer. Histopathology and grade of the tumor may be associated with the aggressiveness of the cancer. Estrogen positivity was seen in 46% of cases which is lower than the Western literature which reported ER positivity of around 60%. PR negativity in 63% of cases is comparable to data from Taucher S et al. [23] which showed PR negativity of 70%. HER2 receptors were found to be positive in 50% of the cases. Triple-negative cancers constituted 31% of cases, which is consistent with many studies showing a higher prevalence of triple-negative breast cancers in the Indian population compared to the Western population [24-26].

In our study, 18 (22%) patients belonging to stage IIB, IIIA on clinical assessment were operable and underwent upfront modified radical mastectomy. Primary surgery was considered in this subset according to the patient's preferences and operable tumors at the initial presentation. The remaining 52 cases (74%) received neoadjuvant chemotherapy followed by modified radical mastectomy based on the downstaging of the tumor. This modality of management has been accepted as a standard procedure for locally advanced inoperable breast cancer as shown by Shenkier et al. [27]. As per the institutional protocol, the majority of the patients received anthracycline-based chemotherapy. In accordance with prevailing NCCN guidelines, 94% of the cases received FEC regimen and the remaining cases received AC and FAC regimens. Buccholz et al. [28] study showed improved outcomes with neoadjuvant chemotherapy followed by surgery. The response was evaluated according to RECIST criteria. Prasad et al. [29] demonstrated that RECIST criteria is more specific than WHO criteria in assessing response to chemotherapy. Although CT and MRI are best in evaluating the response, due to cost considerations and patient willingness, the response was measured clinically using calipers and tape and radiologically by ultrasonography (USG) following each cycle of chemotherapy. Herrada et al. [30] demonstrated that physical examination correlated best with pathological findings in the measurement of the primary tumor. In our study, the median number of cycles was three. In the existing literature, a lot of variation exists in the number of cycles of chemotherapy given in neoadjuvant settings [31]. Investigators have administered either 3-4 cycles of chemotherapy or chemotherapy was continued up to maximal response. Administering chemotherapy up to maximal response is advantageous in a way that if the patient has achieved good CR in less than planned cycles, the continuation of further chemotherapy consolidates the complete response by maintaining the dose intensity. The majority of patients achieved maximal response after three cycles of NACT, few cases of stable and progressive disease received six cycles. 85% of our patients responded well to NACT, which is comparable to the study by Min et al. [16] and Viswambharan JLet al. [22]**,** which showed 86% responders. Complete response was seen in only 9% of cases, which is slightly lower than observed by Taucher S et al. (12%) [23] and Hortobagyi et al. (17%) [31]. Unfortunately, no complete pathological response was seen; this may be attributable to the late stage of presentation. The addition of taxanes and prolonging the cycles could have achieved higher response rates according to National Surgical Adjuvant Breast and Bowel Project (NSABP)trial. In cases that had a partial response, there was a reduction in tumor size of more than 80%. Nonresponders rate (15%) was comparable to Viswambharan JL​​​​​​​et al. [22], Bhattacharya et al. [32], having 13% and 16%, respectively. Identifying responders and nonresponders is important in predicting the survival of the patients, as shown by the studies by Bhattacharya et al. [32] and Deo et al. [33], where disease-free survival improved in responders. 

Various studies have demonstrated that hormone receptor negativity increases the sensitivity to chemotherapy and clinical response rates were higher in hormone receptor-negative cases. Similar to the above observation, in our study, 54% of ER-negative and 68% of PR-negative tumors responded to chemotherapy, with more than 80% reduction in tumor size, which is comparable to other studies [34-36]. Not much difference was noted in HER2/neu-negative tumors, in contrast to many prospective studies showing enhanced response to doxorubicin-based chemotherapy in her2 overexpression tumors. Response with respect to receptor status was not statistically significant individually, but 98% of triple-negative cancers responded well to chemotherapy. In our study, cases belonging to stage IIB (t3n0), IIIA responded well to chemotherapy and hence emphasizes the need for routine screening and awareness among the public regarding signs and symptoms of breast cancer. Out of 32 patients with nodal disease, 21 cases (66%) responded to chemotherapy, which is lower than response rates in primary tumors. In LABC, the prognosis of patients without lymph node metastases is better than those who had lymph node involvement [37-38].

Out of 18 cases that underwent upfront surgery four had margin positivity compared to one case out of 52 cases that received neoadjuvant chemotherapy. The difference was found to be statistically significant (p=0.04). Margin positivity was defined as the presence of in situ or invasive malignancy focally or extensively at the ink of any margin [39]. A study by Yu et al. [39] showed a margin positivity of 10% in cases of breast cancer undergoing mastectomy, in contrast to only 2% in cases that received NACT. Positive margin status has been found to translate into a higher incidence of systemic recurrence, and the presence specifically of a positive deep margin after mastectomy has been associated with an increased local recurrence [39-41]. Hence, NACT should be considered a reasonable alternative in cases of locally advanced operable breast cancer to decrease the rate of margin positivity.

Our study highlights some important challenges in the comprehensive management of breast cancer in developing countries. As pointed out by many studies, the lack of feasible and practical management guidelines, social and health infrastructure differences, and poor access to high-qualitymultimodality treatment facilities are some of the factors resulting in inadequate and inappropriate treatment of breast cancer [8,42]. This was true in our scenario also. Studies have shown that patients with a complete response may be considered for breast-conserving surgery (BCS). Acceptability of BCS depends on the patient's educational and economic background and also on the treating surgeon's training [43]. Due to aforesaid factors, BCS was not incorporated into our treatment protocol. Nevertheless, our study emphasizes the need for multimodality treatment in a protocol-based manner in all tertiary institutes by creating the necessary infrastructure.

Our study has some limitations. Although having an advantage of prospective design, the smaller sample size in our study might have affected the accuracy of our results. Due to logistic reasons and nonavailability in our institute, immunotherapy and radiotherapy could not be incorporated into our study, which might have influenced our results. Detailed evaluation of response variation among molecular subtypes of breast cancer could not be evaluated.

## Conclusions

Locally advanced breast cancer accounted for the predominant number of breast cancer patients, mostly females in their middle age. Neoadjuvant chemotherapy was effective in down staging the tumor in majority of cases, although complete clinical response was lower in our study. Rate of margin positivity in mastectomy specimen can also be reduced if chemotherapy is considered prior to mastectomy. Knowledge of receptor status helps in predicting response to chemotherapy. A protocol based multimodal approach in patients with LABC yields better results.
